# Adrenalectomy in a Patient With Recurrent Hepatocellular Carcinoma in the Adrenal Gland: A Case Report

**DOI:** 10.7759/cureus.45682

**Published:** 2023-09-21

**Authors:** Yoshihiro Mochizuki, Ken Mizokami

**Affiliations:** 1 General Surgery, Tokyo Bay Urayasu Ichikawa Medical Center, Chiba, JPN

**Keywords:** recurrence, prognosis, hepatocellular carcinoma, cancer, alpha-fetoprotein, adrenal gland

## Abstract

Hepatocellular carcinoma recurrence in the adrenal gland is clinically rare, for which there are no clear criteria for examination or treatment. A 70-year-old man underwent laparoscopic low anterior resection seven years prior and was diagnosed with rectal cancer stage 1 (T2N0). Right hepatic resection for suspected hepatocellular carcinoma in his liver six years and nine months prior was performed. Thereafter, the patient was diagnosed with hepatocellular carcinoma stage 3 (T3N0). During the follow-up period, elevated alpha-fetoprotein levels were detected, indicating hepatocellular carcinoma recurrence. Follow-up computed tomography in the delayed phase detected a heterogeneous 5 cm mass in the left adrenal gland. Therefore, we diagnosed the patient with recurrent hepatocellular carcinoma in the adrenal gland, for which we performed an adrenalectomy with a favorable prognosis.

## Introduction

Hepatocellular carcinoma (HCC) is known for its potential to metastasize to various organs, albeit clinically rarely to the adrenal glands. Due to the limited literature and absence of standardized therapeutic approaches, the accumulation of case series and reports is crucial for elucidating the successful management of such cases. Treatment options for adrenal metastasis from HCC include surgical removal [1-4], transcatheter arterial chemoembolization(TACE) [5], radiofrequency ablation (RFA) [6,7], radiation [8], percutaneous ethanol injection therapy (PEIT), or systemic therapy [4,7,9,10]. Herein, we report a case of a single left adrenal metastasis due to recurrence after right hepatectomy for HCC, which was resected with good results.

## Case presentation

**Figure 1:**
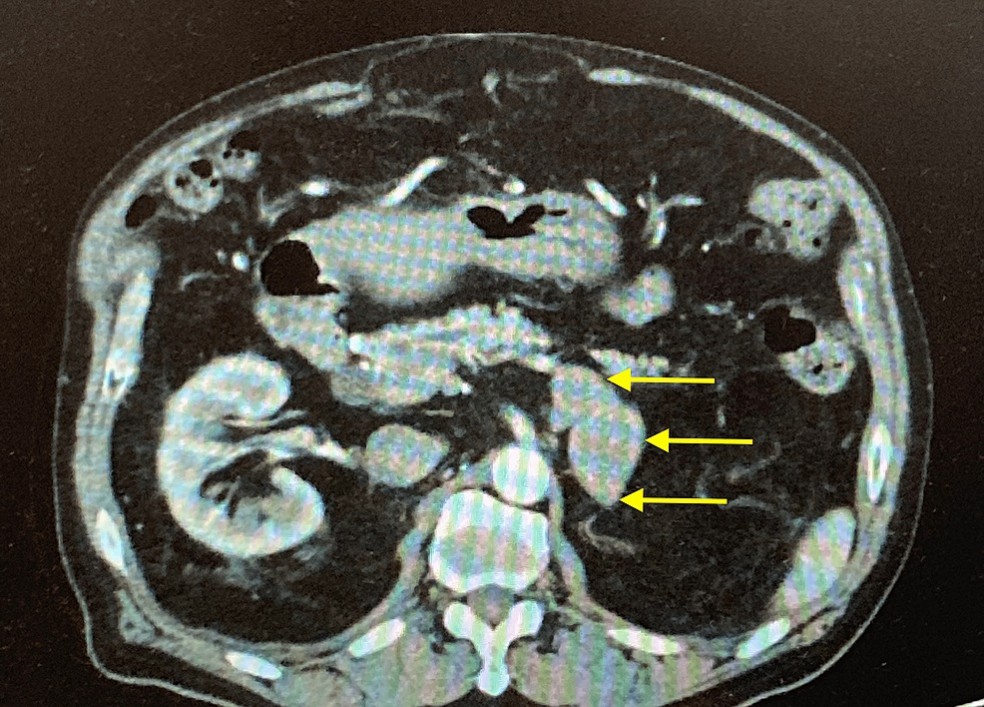
Contrast-enhanced CT of the abdomen Yellow arrows indicate the adrenal tumor measuring 58 mm x 31 mm x 55 mm.

A 70-year-old man with postoperative HCC and rectal carcinoma visited our hospital for surveillance for recurrence. The patient underwent laparoscopic low anterior resection seven years prior, wherein a diagnosis of rectal cancer stage 1 (T2N0) was established. Right hepatic resection was completed six years and nine months prior, after which a diagnosis of HCC stage 3 (T3N0) was made. The patient had an infection of hepatitis B virus that was treated with entecavir. Three months pre-adrenalectomy, follow-up computed tomography (CT) in the delayed phase detected a heterogeneous 5-cm mass in the left adrenal gland (Figure 1). The patient had no history of smoking or drinking alcohol.

Treatment

Elevated levels of alpha-fetoprotein (AFP) were detected (18 ng/mL), leading us to suspect HCC recurrence in the adrenal gland. The left adrenal gland was resected by a laparotomy, with an operating time of 317 min. The drainage tube was inserted into the left lower diaphragm owing to an injured pancreas. This tumor was histopathologically diagnosed as a metastasis from HCC due to the presence of small and large glandular ducts, cord-like arrangements of tumor cells, and biliary-like findings in some areas.

Outcome and follow-up

A postoperative pancreatic fistula was identified because drain amylase was found to be 8019 U/L on postoperative day 3. The patient was followed up without diet until postoperative day 5. The drain was removed on postoperative day 9 following achieving drain amylase levels of 64U/L and an amount of 80 ml per day. The patient was discharged on postoperative day 18. To date, he has survived for 33 months after the procedure.

## Discussion

Adrenal metastasis originating from HCC is clinically rare. In a cohort of 11,770 patients with HCC treated at Yonsei University Medical Center, Park et al. found 45 (0.4%) cases of adrenal metastasis [11]. Abrams et al. reported that 14% of 119 consecutive autopsy cases of colorectal cancer manifested metastasis to the adrenal gland [12]. Given that this is the result of an autopsy, cancer could be considered more advanced and clinically much less common. Although AFP is used to indicate HCC recurrence, the sensitivity is low (approximately 60%) [13]. In addition, whether an elevated AFP could be linked to adrenal gland recurrence remains unknown. In this case, the AFP level was elevated before HCC removal and again at the time of adrenal tumor resection. CT revealed the adrenal tumor in this patient, implying the possibility of rectal cancer recurrence. However, HCC recurrence was diagnosed based on the elevated AFP level. Some case reports indicated that RFA or TACE could be effective in treating adrenal metastasis [6,11,14]. Staubitz et al. revealed that the survival rate following adrenalectomy was superior to that after chemotherapy or local ablation [4]. Other reports indicated that surgery could be a feasible treatment [1,3,14-16]. This case report showed a favorable prognosis after surgery.

## Conclusions

HCC recurrence in the adrenal gland is clinically rare, but we diagnosed adrenal gland metastasis from HCC recurrence preoperatively based on elevated AFP levels correlated with CT findings. Adranarectomy was feasible as the patient tolerated it, and metastasis was limited to the adrenal gland.
